# Long-term metabolic changes with bictegravir/emtricitabine/tenofovir alafenamide or dolutegravir-containing regimens for HIV

**DOI:** 10.1186/s12981-025-00732-w

**Published:** 2025-04-07

**Authors:** Eric S. Daar, Chloe Orkin, Paul E. Sax, Debbie Hagins, Anton Pozniak, Kimberly Workowski, Cynthia Brinson, Juan Manuel Tiraboschi, Hui Liu, Chris Deaton, Cal Cohen, Sharline Madera, Jason T. Hindman, Moti Ramgopal

**Affiliations:** 1 Lundquist Institute at Harbor-UCLA Medical Center, Torrance, CA USA; 2 Queen Mary University of London, London, UK; 3 Brigham and Women’s Hospital, Boston, MA USA; 4 Coastal CARE Centers, Savannah, GA USA; 5 Chelsea and Westminster Hospital, London, UK; 6 London School of Hygiene and Tropical Medicine, London, UK; 7 Emory University, Atlanta, GA USA; 8 Central Health, Austin, TX USA; 9 Hospital Universitari de Bellvitge, Barcelona, Spain; 10 Gilead Sciences, Inc., Foster City, CA USA; 11 Midway Research Center and Midway Specialty Care Center, Fort Pierce, FL USA

**Keywords:** Antiretroviral therapy, Bictegravir, Body mass index, Dolutegravir, Glucose, HIV-1, Tenofovir alafenamide

## Abstract

**Background:**

To evaluate long-term changes in weight and metabolic parameters in people with HIV-1 (PWH) initiating first-line antiretroviral therapy.

**Methods:**

Analysis of two Phase 3, randomized, double-blind, active-controlled trials (1489: NCT02607930; 1490: NCT02607956). PWH received bictegravir/emtricitabine/tenofovir alafenamide (B/F/TAF) or dolutegravir (DTG)-based treatment (Study 1489: dolutegravir/abacavir/lamivudine [DTG/ABC/3TC]; Study 1490: DTG + F/TAF) for 144 weeks, followed by B/F/TAF (96-week open-label extension up to Week 240). Weight and metabolic parameters were assessed through Week 144 by randomized treatment assignment. Weight changes by baseline viral load and CD4 count were evaluated in PWH receiving B/F/TAF from baseline through Week 240. Multivariate modeling explored baseline factors associated with absolute weight and weight change through Week 240 and weight gain ≥ 10% at Week 240.

**Results:**

Median weight and body mass index (BMI) increased over time with B/F/TAF (*n* = 628), DTG/ABC/3TC (*n* = 315), and DTG + F/TAF (*n* = 325). There were no significant differences in change in weight or BMI between the B/F/TAF and DTG + F/TAF groups or between the B/F/TAF and DTG/ABC/3TC groups at Week 144 in either trial, nor were there differences in other metabolic parameters, including incidence of treatment-emergent diabetes mellitus and hypertension through Week 144. Among PWH receiving B/F/TAF (baseline through Week 240), weight increases were greatest soon after initiating antiretroviral therapy (i.e., Weeks 0–48), particularly in participants with baseline viral load > 100,000 copies/ml and/or CD4 count < 200 cells/µl. In multivariate modeling (B/F/TAF pooled data), lower baseline CD4 count and higher HIV-1 RNA were associated with lower baseline weight and greater weight gain, but not absolute weight, from Week 48 through Week 240.

**Conclusions:**

No significant difference in weight change from baseline to Week 144 was found between bictegravir and DTG, or between B/F/TAF and a non-TAF-containing regimen, in these two randomized trials. Furthermore, weight gain following treatment initiation was greatest in the first year of treatment and most pronounced in individuals with more advanced HIV at baseline, supporting the hypothesis that weight gain following initial treatment is linked to a “return to health” in people with advanced HIV.

**Supplementary Information:**

The online version contains supplementary material available at 10.1186/s12981-025-00732-w.

## Background

Metabolic complications, including cardiovascular disease, diabetes mellitus, dyslipidemia, and obesity, are challenges to the long-term management of people with HIV (PWH) [1, 2]. Cardiovascular diseases are reported at higher rates in PWH than in the general population, whereas the precise relationship between HIV and diabetes mellitus remains unclear [3, 4]. Weight gain and obesity are also common in PWH and may contribute to metabolic complications [5–7]; however, some cohort studies have shown rates of weight gain among PWH to be broadly similar to those without HIV [8].

A certain degree of weight gain among PWH who have initiated treatment and achieved virologic suppression is common and may reflect a “return to health”, where alleviation of HIV-associated inflammation and increased catabolism resulting from uncontrolled viral replication may combine with resumption of appetite and restoration of immunity, leading to a return to normal societal weight gain [7]. In support of this, a recent pooled analysis of three large trials indicated that weight gain following antiretroviral therapy (ART) initiation is more marked in PWH with low pretreatment CD4 counts [9].

There is still scrutiny regarding the association between some antiretrovirals, including integrase strand transfer inhibitors (INSTIs) and tenofovir alafenamide (TAF), and changes in lipid and glucose parameters and weight in PWH [7, 10–12]. INSTI-based ART regimens have been associated with weight gain [7, 12–15], and several randomized controlled trials reported larger increases in weight with INSTIs than with non-INSTI regimens, both with and without TAF [7, 16, 17]. These findings have led to concern that INSTIs may promote excessive weight gain [8], and have led to some clinicians and PWH avoiding newer INSTIs and TAF due to uncertainty about effects on weight and metabolic complications [8, 18]. However, a recent expert review of major clinical trials suggested that dolutegravir (DTG), bictegravir (BIC), and TAF may have a neutral influence on weight gain [8]. Further evidence on the metabolic changes that may occur when taking INSTI- and/or TAF-containing regimens is therefore needed to ensure informed decision-making.

Bictegravir/emtricitabine/tenofovir alafenamide (B/F/TAF) is a three-drug, fixed-dose, once-daily complete regimen for the treatment of HIV-1 [19] containing an INSTI, BIC, and two nucleos(t)ide reverse transcriptase inhibitors, emtricitabine and TAF [20–22]. Due to its high barrier to resistance, favorable drug–drug interaction profile, and once-daily dosing without food restrictions, B/F/TAF is recommended in international guidelines (alongside DTG-based therapy) as an initial combination regimen for people initiating ART for the first time, and in switch strategies for PWH who are virologically suppressed [20–22]. Here, we present data comparing metabolic changes in PWH initiating first-line B/F/TAF or DTG-based therapy through 144 weeks during the randomized phase of two Phase 3 trials [23, 24]. We also performed a subanalysis using pooled longer-term data from both studies to investigate weight gain according to viral load and CD4 count at baseline, and multivariate analyses (MVAs) to explore risk factors of (i) absolute weight and change in weight from baseline through Week 240 and (ii) weight gain ≥ 10% at Week 240.

## Methods

### Study design and participants

Studies 1489 (GS-US-380-1489, NCT02607930) and 1490 (GS-US-380-1490, NCT02607956) were Phase 3, randomized, double-blind, multicenter, active-controlled studies (Supplemental Fig. 1). The methods and primary results for both studies have been published previously [23, 24].

Participants were ART-naïve adults (aged ≥ 18 years), with plasma HIV-1 RNA levels ≥ 500 copies/ml (c/ml) at screening and no known resistance to emtricitabine or tenofovir. Eligibility criteria were generally comparable between the two trials, except for the following differences: for Study 1489, individuals with chronic hepatitis B virus (HBV) infection or those who tested positive for human leukocyte antigen (HLA) B*5701 at screening were excluded, and participants had to have an estimated glomerular filtration rate according to the Cockcroft–Gault formula (eGFR_CG_) ≥ 50 ml/min, while for Study 1490, chronic HBV (HBV viral load ≤ 9 log IU/ml) infection was allowed, and participants had to have an eGFR_CG_ ≥30 ml/min.

Participants were randomized 1:1 to receive B/F/TAF or dolutegravir/abacavir/lamivudine (DTG/ABC/3TC; Study 1489), or B/F/TAF or DTG + F/TAF (Study 1490), for ≥ 144 weeks. In both studies, participants who completed the Week 144 visit were invited to participate in an open-label extension (OLE) phase, in which all participants received B/F/TAF for up to 96 weeks (total duration of treatment: ≥240 weeks).

Both studies were undertaken in accordance with the Declaration of Helsinki and were approved by central or site-specific review boards or ethics committees. All participants provided written informed consent.

### Study outcomes and assessments

In both studies, weight, blood pressure, CD4 count, and plasma HIV-1 RNA (Roche TaqMan 2.0; Roche Diagnostics, Rotkreuz, Switzerland) were assessed at Day 1, Weeks 4, 8, and 12, and every 12 weeks thereafter, including during the OLE. Lipid measures (total cholesterol [TC], high-density lipoprotein cholesterol [HDL], low-density lipoprotein cholesterol [LDL], and triglycerides) and fasting glucose levels were assessed after an ≥ 8-hour fast at Day 1, Weeks 12, 24, 48, 72, 96, 120, and 144, and every 12 weeks thereafter in the OLE.

#### As-randomized analyses through Week 144

To assess weight (including ≥ 10% weight gain at Week 144), body mass index (BMI), and metabolic changes through Week 144, safety datasets collected from November 2015 through May 2019 were reviewed. Metabolic outcomes included treatment-emergent metabolic comorbidities (diabetes mellitus, hypertension) and changes in fasting blood glucose levels, fasting lipid parameters, and BMI. Outcomes were based on adverse event reporting using Standardized Medical Dictionary for Regulatory Activities (MedDRA) Query (SMQ) search lists. Diabetes mellitus was defined as hyperglycemia/newonset diabetes mellitus SMQ (narrow scope) in MedDRA version 23.1. Hypertension was defined as hypertension SMQ. Participants with a medical history of diabetes mellitus and hypertension were excluded from assessments of treatment-emergent diabetes or hypertension, respectively.

#### Analyses through Week 240

To assess long-term changes in weight and virologic outcomes in Studies 1489 and 1490, safety datasets collected from November 2015 through July 2021 were reviewed. Pooled data from participants who were initially randomized to receive B/F/TAF in either trial were included in the pooled B/F/TAF group and used to analyze virologic suppression (HIV-1 RNA < 50 c/ml by missing = excluded and missing = failure analyses) and change in CD4 count, stratified according to baseline viral load and CD4 count, ≥ 10% weight change up to Week 240, and in MVA of absolute weight and weight change. Data from a subset of participants in the pooled B/F/TAF group with available data at baseline and Week 240 were used to assess weight change and absolute weight, stratified according to baseline viral load and CD4 count, and for MVA of risk factors associated with ≥ 10% weight change at Week 240. Stratification categories were as follows: viral load ≤ 100,000 c/ml or > 100,000 c/ml; CD4 count < 200 or ≥ 200 cells/µl; viral load > 100,000 c/ml and CD4 count < 200 cells/µl.

### Statistical analyses

Weight and other metabolic analyses were not prespecified and thus are *post hoc*. All data through Week 144 were analyzed according to randomized treatment group; data through Week 240 for those assigned to B/F/TAF were pooled between the two trials. Demographics and baseline characteristics, hypertension, fasting lipid parameters, and change from baseline in BMI and weight were summarized using descriptive statistics. For participants with ≥ 10% weight gain at Week 240, differences between baseline characteristics categories were analyzed using the Cochran–Mantel–Haenszel test for categorical data (sex at birth, race, HIV-1 RNA, CD4 count) or the Wilcoxon rank sum test for continuous data (age, weight, BMI). In each trial, differences between treatment groups were compared using the Fisher exact test for categorical data (proportion of participants with ≥ 10% weight gain at Week 144) or the 2-sided Wilcoxon rank sum test for continuous data (fasting blood glucose, changes from baseline in BMI and weight).

Linear regression models were used to explore baseline risk factors associated with weight and weight change at Weeks 48, 96, 144, 196, and 240. For multivariate models of weight and weight change at each visit, stepwise selections were used to identify risk factors for inclusion in models (Supplemental Methods). A separate stepwise model selection identified baseline risk factors associated with ≥ 10% weight increase at Week 240 using logistic regression models (Supplemental Methods). All analyses were performed using SAS version 9.4 (SAS Institute, Cary, NC, USA).

## Results

### Participant characteristics and study treatment

The disposition of participants is shown in Supplemental Fig. 2. In Study 1489, 314 and 315 participants were randomized to receive B/F/TAF or DTG/ABC/3TC, respectively, and are included in this analysis. In Study 1490, 320 and 325 participants were randomized to receive B/F/TAF or DTG + F/TAF, respectively. Six participants in the B/F/TAF treatment group of Study 1490 did not have postbaseline data available and were excluded from the analyses of weight. Overall, 506 participants from the B/F/TAF treatment groups across both studies received B/F/TAF in the OLE (Supplemental Fig. 2).

Baseline characteristics of participants in both studies, shown in Supplemental Table 1, were generally balanced across treatment groups and studies. The median HIV-1 RNA levels ranged from 4.4 to 4.5 log_10_ c/ml across treatment groups; median CD4 count ranged from 440 to 450 cells/µl. The majority of PWH (≥ 89%) were asymptomatic, and median BMI was 25 kg/m^2^ in all treatment groups and studies.

### As-randomized analyses through Week 144

**Fig. 1:**
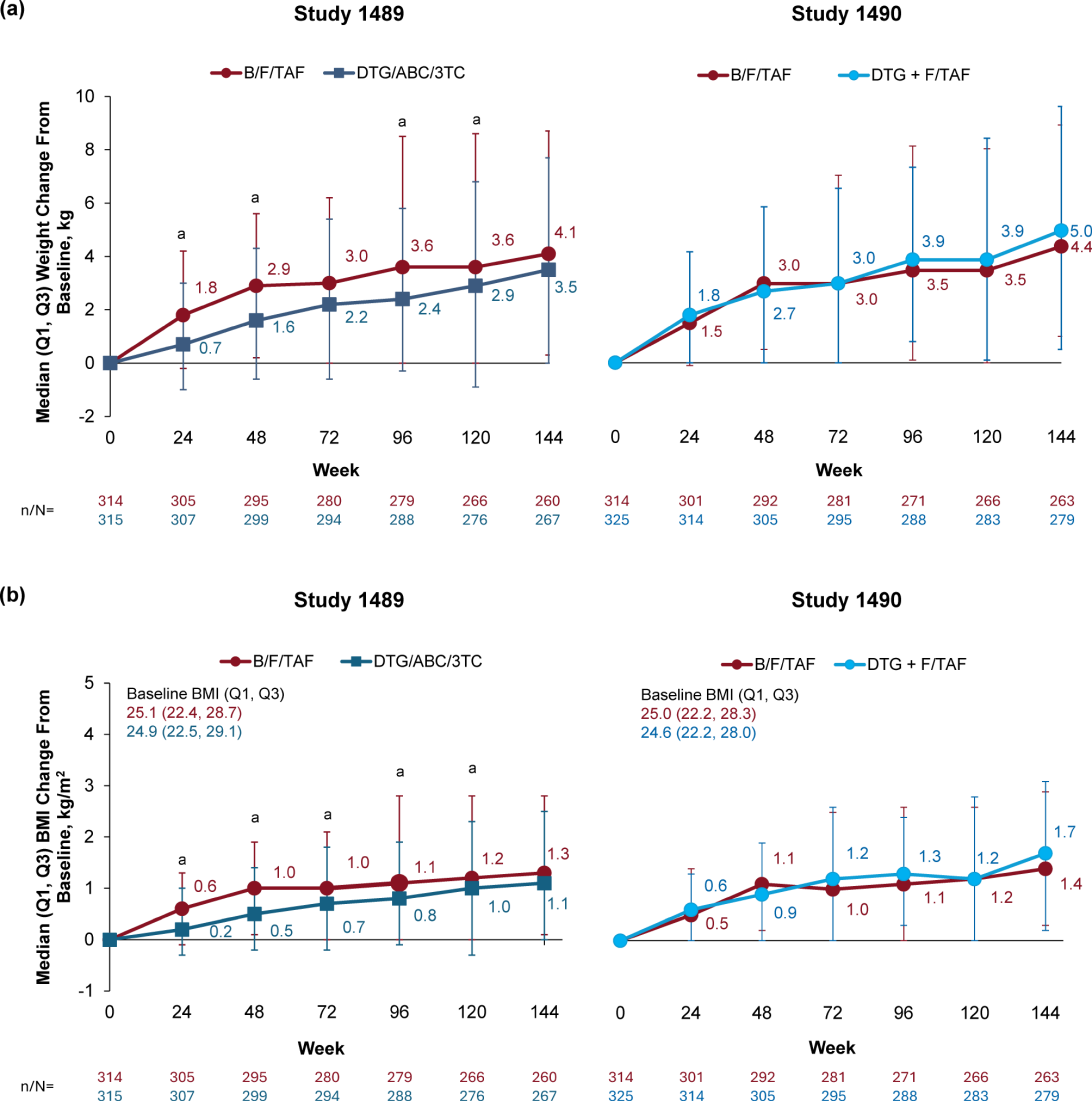
Change from baseline in (**a**) weight and (**b**) BMI through Week 144 in Study 1489 and Study 1490. ^a^*p* < 0.05 from 2-sided Wilcoxon rank sum test to compare two treatment groups in each study. 3TC, lamivudine; ABC, abacavir; B, bictegravir; BMI, body mass index; DTG, dolutegravir; F, emtricitabine; Q, quartile; TAF, tenofovir alafenamide

Median weight and BMI changes over time by randomized group assignment for both trials are shown in Fig. 1. Both median weight and BMI increased over time in all treatment groups in both studies. In each trial, there was no statistically significant difference in change from baseline in weight or BMI at Week 144 in participants randomized to B/F/TAF versus DTG-based regimens (DTG/ABC/3TC in Study 1489 and DTG + F/TAF in Study 1490); the difference in median weight change between treatment groups ranged from 0.6 to 1.3 kg (Fig. 1A). The proportion of participants with ≥ 10% weight gain at Week 144 was similar between B/F/TAF and DTG/ABC/3TC (29.2% [76/260] and 24.7% [66/267], respectively; *p* = 0.28), and between B/F/TAF and DTG + F/TAF (30.4% [80/263] and 31.9% [89/279], respectively; *p* = 0.71).

**Fig. 2:**
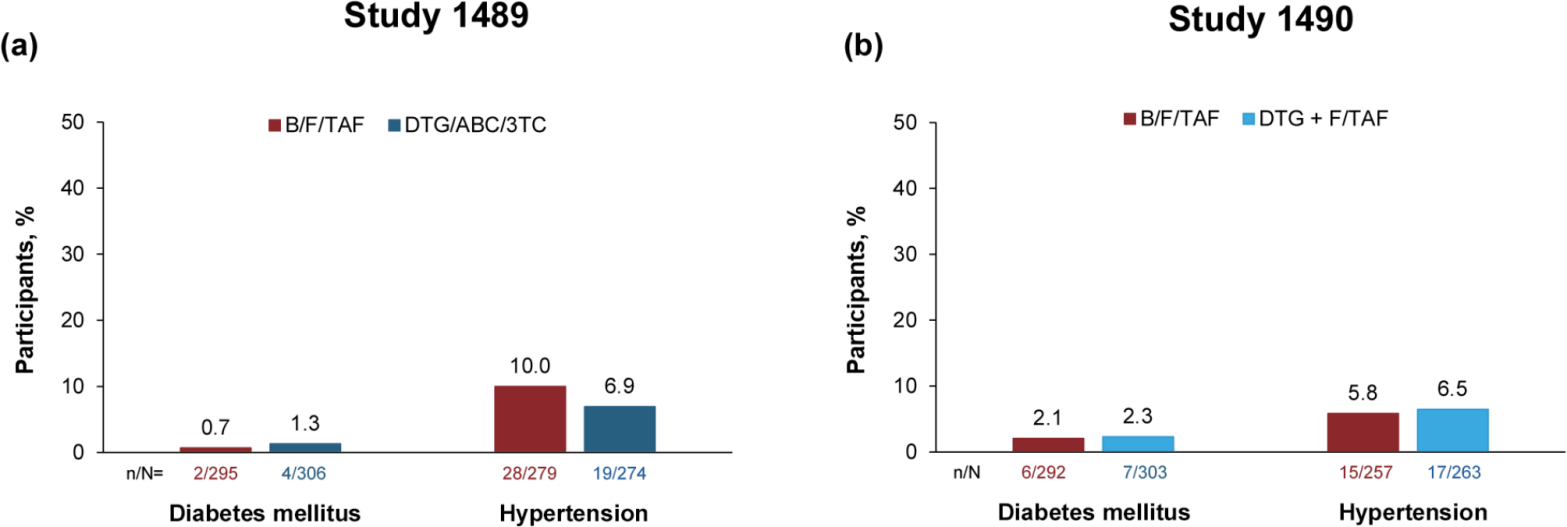
Treatment-emergent diabetes mellitus^a^ and hypertension^b^ through Week 144 in (**a**) Study 1489 and (**b**) Study 1490. ^a^Participants with medical history of diabetes mellitus were excluded. ^b^Participants with medical history of hypertension were excluded; events defined using search list “hyperglycemia/new onset diabetes mellitus (SMQ)– narrow scope” and “hypertension (SMQ)” in MedDRA version 23.1. 3TC, lamivudine; ABC, abacavir; B, bictegravir; DTG, dolutegravir; F, emtricitabine; MedDRA, Medical Dictionary for Regulatory Activities; SMQ, Standardized MedDRA Query; TAF, tenofovir alafenamide

Treatment-emergent diabetes mellitus occurred in 1.6% (19/1196) of participants, and treatment-emergent hypertension occurred in 7.4% (79/1073) of participants, across both studies (Fig. 2). The incidence of both conditions was similar between the B/F/TAF (diabetes mellitus: 0.7%; hypertension: 10.0%) and DTG/ABC/3TC (diabetes mellitus: 1.3%; hypertension: 6.9%) groups (Study 1489), and the B/F/TAF (diabetes mellitus: 2.1%; hypertension: 5.8%) and DTG + F/TAF (diabetes mellitus: 2.3%; hypertension: 6.5%) groups (Study 1490).

Median fasting blood glucose levels remained relatively stable over time in all treatment groups, and median changes from baseline at Week 144 were not significantly different when compared between treatment groups (Study 1489: *p* = 0.64; Study 1490: *p* = 0.96; Supplemental Fig. 3).

**Fig. 3:**
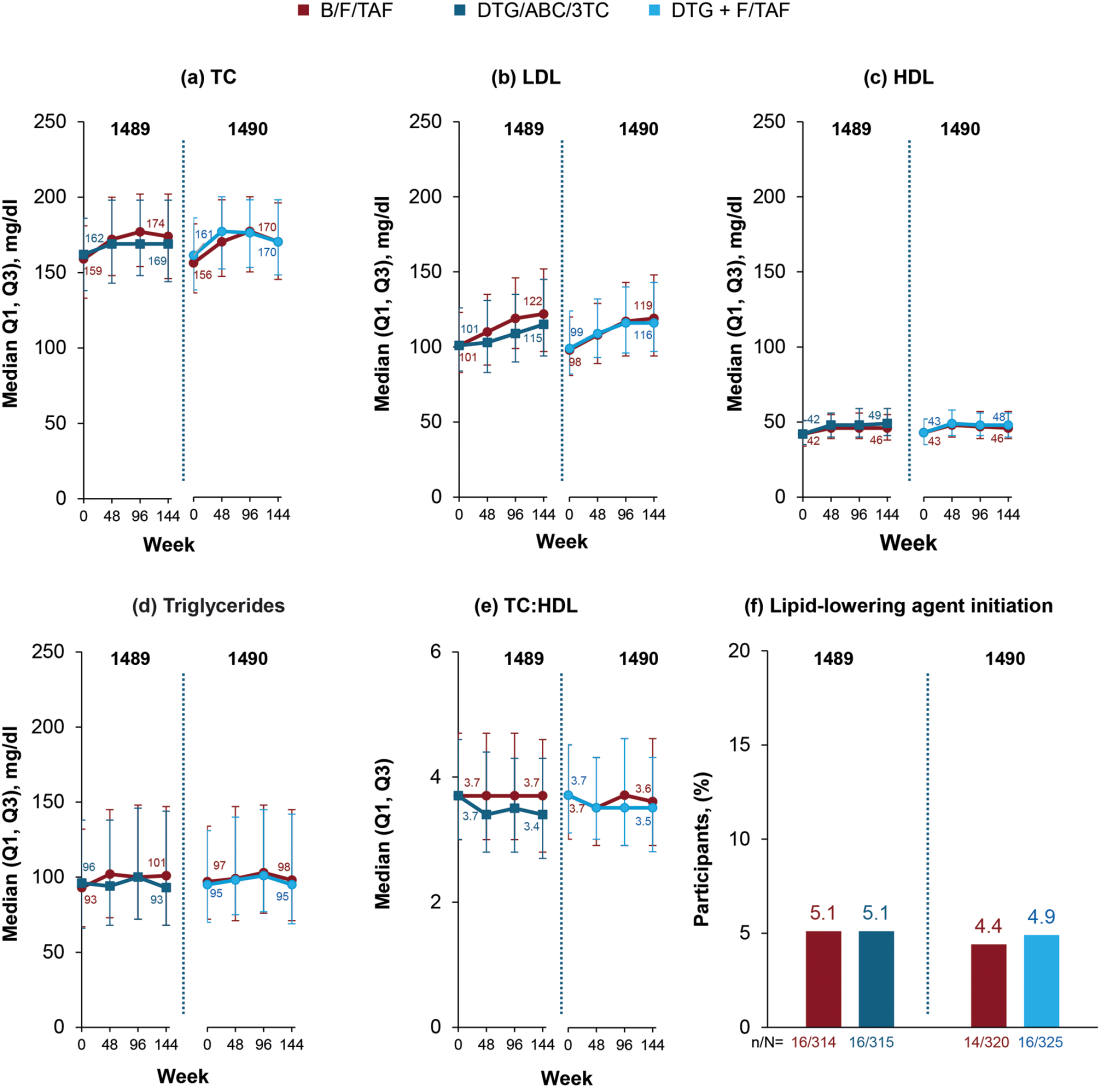
(**a**–**e**) Fasting lipid parameters and (**f**) initiation of lipid-lowering agents through Week 144 in Study 1489 and Study 1490. 3TC, lamivudine; ABC, abacavir; B, bictegravir; DTG, dolutegravir; F, emtricitabine; HDL, high-density lipoprotein cholesterol; LDL, low-density lipoprotein cholesterol; Q, quartile; TAF, tenofovir alafenamide; TC, total cholesterol; TC: HDL, total cholesterol to high-density lipoprotein cholesterol ratio

Fasting lipid parameters were similar between treatment groups through Week 144, with minor increases from baseline observed in all groups (Fig. 3). The proportions of participants who initiated lipid-lowering therapy were also similar across treatment groups, and the impact of all three regimens on the TC:HDL ratio was minimal. Similar percentages of participants in each group in both studies experienced treatment-emergent graded laboratory abnormalities in fasting lipid parameters (Supplemental Fig. 4).

### Analyses through Week 240

Baseline characteristics of the pooled B/F/TAF group, stratified by baseline viral load and CD4 count, are shown in Supplemental Table 2. Participants with a baseline viral load > 100,000 c/ml, CD4 count < 200 cells/µl, or both, were slightly older and less likely to be asymptomatic compared with participants in the other groups.

#### Analysis of change in weight

**Fig. 4:**
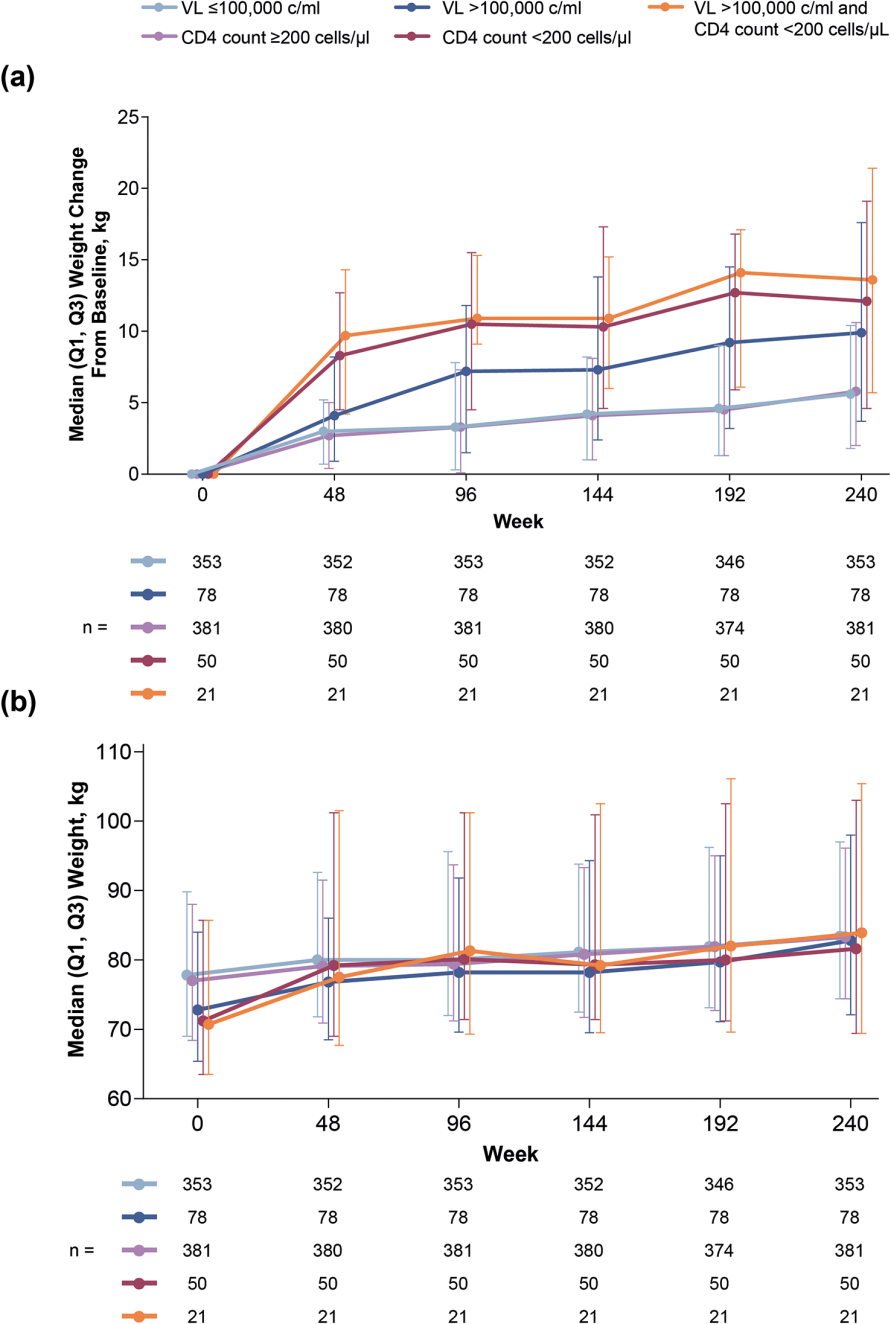
(**a**) Weight change from baseline over time and (**b**) absolute weight according to baseline viral load and CD4 count among participants receiving B/F/TAF up to Week 240 (pooled B/F/TAF groups). Includes participants with weight data at baseline and Week 240. B, bictegravir; c, copies; F, emtricitabine; Q, quartile; TAF, tenofovir alafenamide; VL, viral load

Weight change over 240 weeks of B/F/TAF treatment according to baseline viral load and CD4 count is shown in Fig. 4A. The greatest weight gain was observed in participants with the highest viral load and lowest CD4 count at baseline, with the largest weight changes occurring in the first 48 weeks of treatment, followed by smaller weight changes every year thereafter. Between Weeks 48 and 240, weight change was similar for all groups, irrespective of baseline viral load and CD4 count.

Multivariate regression modeling of weight change at Week 48through Week 240 (Supplemental Table 3) confirmed that the lower baseline CD4 count category (< 200 cells/µl) was strongly associated with weight gain at Week 48 and every timepoint thereafter up to Week 240. Except for Week 48, the higher baseline viral load category (> 100,000 c/ml) was significantly associated with weight gain at all timepoints. Higher baseline BMI and more advanced HIV disease status (AIDS or symptomatic) were also associated with weight gain at earlier timepoints.

#### Analysis of absolute weight

Participants with baseline viral load > 100,000 c/ml and/or CD4 count < 200 cells/µl at baseline had lower baseline weights. By Week 48, their weight was comparable to those who initiated B/F/TAF without high viral load or low CD4 cell count at baseline (range: 76.8–80.0 kg; Fig. 4B). This was consistent with multivariate linear regression modeling (Table 1), which identified lower CD4 count and higher viral load as independent risk factors for lower baseline weight, alongside Hispanic ethnicity, non-Black race, female sex, and younger age. From Week 48, baseline CD4 count and HIV-1 RNA levels were no longer associated with absolute weight, unlike Hispanic ethnicity, non-Black race, and female sex, which remained significant at most timepoints.

**Table 1:** Effect of baseline characteristics on absolute weight at baseline and Weeks 48–240

	Estimated mean (95% CI) weight difference, kg *p* value					
Variable	Baseline	Week				
		48	96	144	192	240
Ethnicity (non-Hispanic vs. Hispanic)	**6.1 (2.8, 9.5) ** ** < 0.001**	**6.7 (2.8, 10.5)** ** < 0.001**	**6.1 (2.1, 10.2)** **0.003**	**6.1 (1.8, 10.3)** **0.005**	**7.5 (3.0, 11.9)** **0.001**	**7.6 (2.7, 12.4)** **0.002**
Race (Black vs. non-Black)	**5.2 (2.1, 8.2)** **0.001**	**6.3 (2.8, 9.9)** ** < 0.001**	**6.6 (2.8, 10.3)** ** < 0.001**	**7.0 (3.1, 11.0)** ** < 0.001**	**5.8 (1.7, 9.9)** **0.006**	**6.3 (1.8, 10.9)** **0.007**
Sex at birth (male vs. female)	**6.3 (1.9, 10.7)** **0.005**	**6.0 (0.9, 11.0)** **0.020**	5.1 (−0.4, 10.5) 0.068	5.9 (−0.02, 11.9) 0.051	**6.4 (0.2, 12.6)** **0.045**	**7.0 (0.3, 13.6)** **0.040**
Age (< 50 vs. ≥50 years)	**−4.8 (−8.6, −1.0)** **0.013**	−3.7 (−8.1, 0.7) 0.097	−3.1 (−7.8, 1.5) 0.183	−3.8 (−8.7, 1.0) 0.121	−3.2 (−8.3, 1.9) 0.217	−2.6 (−8.2, 3.0) 0.358
CD4 count (< 200 vs. ≥200 cells/µl)	**−6.7 (−11.5, −1.9)** **0.006**	−2.0 (−7.5, 3.6) 0.487	−1.9 (−7.8, 4.0) 0.526	−1.9 (−8.0, 4.2) 0.548	−2.0 (−8.4, 4.4) 0.539	−3.0 (−10.2, 4.2) 0.414
HIV-1 RNA (> 100,000 vs. ≤100,000 c/ml)	**−4.3 (−7.9, −0.7)** **0.021**	−3.7 (−7.9, 0.6) 0.093	−2.8 (−7.4, 1.7) 0.218	−2.1 (−6.8, 2.6) 0.386	−2.2 (−7.0, 2.6) 0.371	−2.4 (−7.8, 2.9) 0.375
HIV disease status (asymptomatic vs. AIDS or symptomatic)	2.6 (−2.6, 7.8) 0.330	5.0 (−1.1, 11.0) 0.109	6.3 (−0.2, 12.9) 0.059	5.2 (−1.7, 12.0) 0.137	5.6 (−1.4, 12.6) 0.114	6.5 (−1.5, 14.4) 0.109

Differences in mean weight, 95% CIs, and *p* values were derived from a multivariate linear regression model for absolute weight at each timepoint (baseline and Week 48 to Week 240). Bold font indicates statistical significance (*p* < 0.05). Baseline value was defined as the last nonmissing value obtained on or prior to the first dose of the study treatment (B/F/TAF). B, bictegravir; CI, confidence interval; F, emtricitabine; TAF, tenofovir alafenamide

#### Analysis of ≥ 10% weight gain

To understand factors associated with more clinically relevant weight gain, we stratified pooled participant data into those gaining ≥ 10% of their body weight at Week 240 versus those gaining < 10%. At Week 240, 40.6% of participants receiving B/F/TAF experienced ≥ 10% weight gain (median weight gain: 16.9%), while at any timepoint up to Week 240, 52.2% of participants receiving B/F/TAF experienced ≥ 10% weight gain.

In multivariate logistic regression modeling (Supplemental Table 4), a lower baseline CD4 count, higher baseline viral load, and baseline BMI categorized as underweight/normal were significant risk factors for greater odds of ≥ 10% weight gain at Week 240, compared with a higher CD4 count, lower viral load, and BMI classed as overweight, respectively.

#### Efficacy analysis

Efficacy outcomes up to Week 240 have been published previously [25]. In the current analysis, virologic suppression occurred in the first 48 weeks for most participants in all viral load and CD4 count groups (Supplemental Fig. 5), coupled with a rapid increase in CD4 count through Week 48 (Supplemental Fig. 6), mirroring the time-course of weight gain.

## Discussion

The evaluation of metabolic outcomes and assessment of weight gain following initiation of ART is of interest, as some PWH and their providers may be hesitant to start INSTI- or TAF-based treatments believed to cause excessive weight gain or place PWH at an unnecessary risk of diabetes or hyperlipidemia. Using data from two Phase 3, randomized, double-blind clinical trials [23, 24], we have shown that changes in weight, as well as blood glucose and lipid parameters, in intention-to-treat analyses through Week 144 are similar across three recommended ART regimens (including a comparison of BIC to DTG and of B/F/TAF to a regimen without TAF).

Weight and BMI increased over the 144-week study period for most participants, irrespective of the ART regimen initiated (i.e., B/F/TAF, DTG/ABC/3TC, or DTG + F/TAF), as would be expected in PWH who have not previously received ART. In the individual trials, no statistically significant difference was observed at any timepoint in change in weight or BMI between the B/F/TAF and DTG + F/TAF regimens, and since the only difference between these treatments is the INSTI, this would indicate no difference between BIC and DTG. In the trial comparing B/F/TAF to a non-TAF-containing regimen, no statistically significant difference at Week 144 was seen in weight or BMI, although differences were observed at earlier timepoints, as has been seen previously in studies of ABC- versus TAF-containing ART [16]. We observed similar rates of treatment-emergent hypertension, diabetes mellitus, and fasting lipid abnormalities, and in fasting glucose and lipid parameters, between treatment groups, indicating that the initial difference in weight gain with B/F/TAF versus DTG/ABC/3TC was not associated with differences in rates of metabolic complications.

To further investigate weight gain in PWH initiating B/F/TAF as first-line therapy, we used pooled B/F/TAF data from both studies over 240 weeks to investigate weight gain according to viral load and CD4 count at baseline. In participants who were initially randomized to B/F/TAF and continued through 5 years of follow-up, there were greater median weight changes in the first year for participants with high viral load and/or low CD4 count at baseline, compared with the low viral load or high CD4 count groups. This may be because those with the most advanced disease have had the most HIV disease–related weight loss prior to ART initiation and, consequently, have the largest amount of weight to gain during the first year of treatment as part of returning to health [9], also supported by the fact that after Week 48, participants across all groups had similar median absolute weights. The smaller weight increases observed between Weeks 96–144, 144–192, and 192–240 (Fig. 4A) support the hypothesis of a return to pre-HIV weight and resumption of normal societal weight fluctuations. Importantly, the largest changes in weight in individuals with higher viral loads or lower CD4 counts at baseline occurred during the first year of the study, in parallel with reductions in viral load and increases in CD4 count.

In our study, baseline CD4 count category was significantly associated with change in weight at Week 48 and all subsequent timepoints through Week 240 and was a stronger risk factor than baseline viral load, which was also significantly associated with weight change at Week 96 and thereafter. Furthermore, baseline viral load category and CD4 category were statistically significant risk factors associated with lower baseline weight, but not absolute weight from Week 48 through Week 240. This indicates that the weight gain observed in participants with advanced disease in the first year of treatment likely resulted from alleviation of HIV disease–related weight loss. Our findings are supported by a recent pooled analysis of three first-line ART trials (ADVANCE, NAMSAL, and WHRI001; *N* = 2202), which found that participants with baseline CD4 counts < 100 cells/µl had the largest weight gain during ART treatment [9], and by an MVA of weight gain after initiation of ART in PWH, which identified lower CD4 count, higher HIV-1 RNA, lower BMI, female sex, and Black race as risk factors for ≥ 10% weight gain through Week 48 [7].

As expected for a population receiving ART for the first time, a substantial proportion of participants experienced ≥ 10% weight gain over 240 weeks. Notably, the proportions of participants with ≥ 10% weight gain were similar between B/F/TAF and DTG-based comparator groups at the end of the blinded phase at Week 144 in each trial, indicating no difference between BIC- and DTG-based therapy. Furthermore, our results show that some individuals experienced ≥ 10% weight gain and subsequently lost weight, as indicated by the greater proportion of participants taking B/F/TAF with ≥ 10% weight gain at any timepoint up to Week 240 (52%) versus at Week 240 (41%), underscoring the role that individuals and their clinicians can play in effectively managing weight gain regardless of ART.

This analysis does have some limitations. The median age at baseline was relatively low, and there was only a small number of participants with advanced HIV–related immunosuppression, and a small proportion of female and non-White participants. Future studies should examine changes in body composition or other measures as a more reliable marker of adiposity-related risks [26], as well as other factors that could contribute to weight change, such as dietary intake, concomitant medications, and activity levels.

## Conclusions

These findings provide important information regarding the effect of B/F/TAF and DTG-based therapies on weight gain in PWH and highlight the low incidence of treatment-emergent diabetes mellitus and hypertension. This new evidence adds further support to the hypothesis that weight gain after starting ART is linked to a return to health in people with more advanced HIV.

## Electronic supplementary material

Below is the link to the electronic supplementary material.

Supplementary Material 1

## Data Availability

Gilead Sciences shares anonymized individual patient data upon request or as required by law or regulation with qualified external researchers based on submitted curriculum vitae and reflecting non-conflict of interest. The request proposal must also include a statistician. Approval of such requests is at Gilead Science’s discretion and is dependent on the nature of the request, the merit of the research proposed, the availability of the data and the intended use of the data. Data requests should be sent to Data.Sharing@gilead.com.

## Declarations

### Prior presentation

Data were partially previously presented as an oral presentation at IDWeek 2021 (virtual), September 29–October 3, and in poster format at IDWeek 2022, October 19–23, Washington, DC, USA.

## Abbreviations

3TC: Lamivudine
ABC: Abacavir
AIDS: Acquired immunodeficiency syndrome
ART: Antiretroviral therapy
B / BIC: Bictegravir
BL: Baseline
BMI: Body mass index
c: Copies
CI: Confidence interval
DTG: Dolutegravir
eGFR_CG_: Estimated glomerular filtration rate according to the Cockcroft–Gault formula
F / FTC: Emtricitabine
HBV: Hepatitis B virus
HCV: Hepatitis C virus
HDL: High-density lipoprotein cholesterol
HLA: Human leukocyte antigen
INSTI: Integrase strand transfer inhibitor
LDL: Low-density lipoprotein cholesterol
MedDRA: Medical Dictionary for Regulatory Activities
OLE: Open-label extension
PWH: People with HIV-1
qd: Once daily
SMQ: Standardized MedDRA Query
TAF: Tenofovir alafenamide
TC: Total cholesterol
TFV: Tenofovir
VL: Viral load

## References

[1] Cummins NW. Metabolic complications of chronic HIV infection: a narrative review. Pathogens. 2022;11(2):197. 35215140 10.3390/pathogens11020197 PMC8879342

[2] Henning RJ, Greene JN. The epidemiology, mechanisms, diagnosis and treatment of cardiovascular disease in adult patients with HIV. Am J Cardiovasc Dis. 2023;13(2):101–21. 37213313 PMC10193251

[3] Hernandez-Romieu AC, Garg S, Rosenberg ES, Thompson-Paul AM, Skarbinski J. Is diabetes prevalence higher among HIV-infected individuals compared with the general population? Evidence from MMP and NHANES 2009–2010. BMJ Open Diabetes Res Care. 2017;5(1):e000304. 28191320 10.1136/bmjdrc-2016-000304 PMC5293823

[4] Shah ASV, Stelzle D, Lee KK, Beck EJ, Alam S, Clifford S, et al. Global burden of atherosclerotic cardiovascular disease in people living with HIV: systematic review and meta-analysis. Circulation. 2018;138(11):1100–12. 29967196 10.1161/CIRCULATIONAHA.117.033369 PMC6221183

[5] Bailin SS, Gabriel CL, Wanjalla CN, Koethe JR. Obesity and weight gain in persons with HIV. Curr HIV/AIDS Rep. 2020;17(2):138–50. 32072466 10.1007/s11904-020-00483-5 PMC7719267

[6] Herrin M, Tate JP, Akgun KM, Butt AA, Crothers K, Freiberg MS, et al. Weight gain and incident diabetes among HIV-infected veterans initiating antiretroviral therapy compared with uninfected individuals. J Acquir Immune Defic Syndr. 2016;73(2):228–36. 27171741 10.1097/QAI.0000000000001071 PMC5023454

[7] Sax PE, Erlandson KM, Lake JE, McComsey GA, Orkin C, Esser S, et al. Weight gain following initiation of antiretroviral therapy: risk factors in randomized comparative clinical trials. Clin Infect Dis. 2020;71(6):1379–89. 31606734 10.1093/cid/ciz999 PMC7486849

[8] Wohl DA, Koethe JR, Sax PE, McComsey GA, Kuritzkes DR, Moyle G, et al. Antiretrovirals and weight change: weighing the evidence. Clin Infect Dis. 2024;79(4):999–1005. 38606799 10.1093/cid/ciae191

[9] Hill A, Tovar Sanchez T, Delaporte E, Sokhela S, Simmons B, Kouanfack C, et al. Low CD4 counts predict excessive weight gains during first-line treatment for HIV. J Antimicrob Chemother. 2024;79(9):2369–2378. 39028639 10.1093/jac/dkae238

[10] Galdamez R, Garcia JA, Fernandez M, Robledano C, Agullo V, Garcia-Abellan J, et al. Short-term increase in risk of overweight and concomitant systolic blood pressure elevation in treatment-naive persons starting INSTI-based antiretroviral therapy. Open Forum Infect Dis. 2019;6(12):ofz491. 32128334 10.1093/ofid/ofz491 PMC7047949

[11] Lagathu C, Bereziat V, Gorwood J, Fellahi S, Bastard JP, Vigouroux C, et al. Metabolic complications affecting adipose tissue, lipid and glucose metabolism associated with HIV antiretroviral treatment. Expert Opin Drug Saf. 2019;18(9):829–40. 31304808 10.1080/14740338.2019.1644317

[12] Pantazis N, Sabin CA, Grabar S, Van der Valk M, Jarrin I, van Sighem A, et al. Changes in bodyweight after initiating antiretroviral therapy close to HIV-1 seroconversion: an international cohort collaboration. Lancet HIV. 2024;11(10):e660–9. 39186940 10.1016/S2352-3018(24)00183-8

[13] Bakal DR, Coelho LE, Luz PM, Clark JL, De Boni RB, Cardoso SW, et al. Obesity following ART initiation is common and influenced by both traditional and HIV-/ART-specific risk factors. J Antimicrob Chemother. 2018;73(8):2177–85. 29722811 10.1093/jac/dky145 PMC6054231

[14] Bourgi K, Rebeiro PF, Turner M, Castilho JL, Hulgan T, Raffanti SP, et al. Greater weight gain in treatment-naive persons starting dolutegravir-based antiretroviral therapy. Clin Infect Dis. 2020;70(7):1267–74. 31100116 10.1093/cid/ciz407 PMC8205610

[15] Lahiri CD, Xu Y, Wang K, Alvarez JA, Sheth AN, O’Halloran J, et al. Weight and body mass index change after switching to integrase inhibitors or tenofovir alafenamide among women living with HIV. AIDS Res Hum Retroviruses. 2021;37(6):461–7. 33231474 10.1089/aid.2020.0197 PMC8213005

[16] Erlandson KM, Carter CC, Melbourne K, Brown TT, Cohen C, Das M, et al. Weight change following antiretroviral therapy switch in people with viral suppression: pooled data from randomized clinical trials. Clin Infect Dis. 2021;73(8):1440–51. 33987636 10.1093/cid/ciab444 PMC12097995

[17] Venter WDF, Moorhouse M, Sokhela S, Fairlie L, Mashabane N, Masenya M, et al. Dolutegravir plus two different prodrugs of tenofovir to treat HIV. N Engl J Med. 2019;381(9):803–15. 31339677 10.1056/NEJMoa1902824

[18] Maartens G, Sinxadi P, Venter WDF. Weight gain on dolutegravir: association is not the same as causation. South Afr J HIV Med. 2023;24(1):1500. 37293606 10.4102/sajhivmed.v24i1.1500 PMC10244923

[19] Gilead Sciences. Biktarvy Prescribing information. 2024. https://www.gilead.com/~/media/files/pdfs/medicines/hiv/biktarvy/biktarvy_pi.pdf

[20] European AIDS, October Clinical Society. Guidelines version 12. 2023. https://www.eacsociety.org/media/guidelines-12.0.pdf

[21] Gandhi RT, Bedimo R, Hoy JF, Landovitz RJ, Smith DM, Eaton EF, et al. Antiretroviral drugs for treatment and prevention of HIV infection in adults: 2022 recommendations of the international antiviral Society-USA panel. JAMA. 2023;329(1):63–84. 36454551 10.1001/jama.2022.22246

[22] Panel on Antiretroviral Guidelines for Adults and Adolescents. Guidelines for the use of antiretroviral agents in adults and adolescents with HIV. Department of health and human services. September 2024. https://clinicalinfo.hiv.gov/en/guidelines/adult-and-adolescent-arv

[23] Gallant J, Lazzarin A, Mills A, Orkin C, Podzamczer D, Tebas P, et al. Bictegravir, emtricitabine, and tenofovir alafenamide versus dolutegravir, abacavir, and lamivudine for initial treatment of HIV-1 infection (GS-US-380-1489): a double-blind, multicentre, phase 3, randomised controlled non-inferiority trial. Lancet. 2017;390(10107):2063–72. 28867497 10.1016/S0140-6736(17)32299-7

[24] Sax PE, Pozniak A, Montes ML, Koenig E, DeJesus E, Stellbrink HJ, et al. Coformulated bictegravir, emtricitabine, and tenofovir alafenamide versus dolutegravir with emtricitabine and tenofovir alafenamide, for initial treatment of HIV-1 infection (GS-US-380-1490): a randomised, double-blind, multicentre, phase 3, non-inferiority trial. Lancet. 2017;390(10107):2073–82. 28867499 10.1016/S0140-6736(17)32340-1

[25] Sax PE, Arribas JR, Orkin C, Lazzarin A, Pozniak A, DeJesus E, et al. Bictegravir/emtricitabine/tenofovir Alafenamide as initial treatment for HIV-1: five-year follow-up from two randomized trials. EClinicalMedicine. 2023;59:101991. 37200995 10.1016/j.eclinm.2023.101991 PMC10186485

[26] De Lorenzo A, Bianchi A, Maroni P, Iannarelli A, Di Daniele N, Iacopino L, et al. Adiposity rather than BMI determines metabolic risk. Int J Cardiol. 2013;166(1):111–7. 22088224 10.1016/j.ijcard.2011.10.006

