# Supplementary material for: Long-term metabolic changes with bictegravir/emtricitabine/tenofovir alafenamide or dolutegravir-containing regimens for HIV
Source: AIDS Res Ther. 2025 Apr 7;22:45. doi: 10.1186/s12981-025-00732-w (PMC11978100; doi:10.1186/s12981-025-00732-w)
Supplement: Supplementary file 1 — Supplementary Material 1 [file 12981_2025_732_MOESM1_ESM.pdf]

Additional file 1

Supplemental Methods

Multivariate regression model for weight change from baseline

Stepwise model selection was used to identify baseline risk factors associated with change from baseline in weight using linear regression models among all postbaseline visits, which included nine baseline risk factors. The baseline risk factors considered for inclusion were six traditional obesity risk factors: age (<50 vs ≥ 50 years), sex at birth (male vs female), race (Black vs non-Black), ethnicity (Hispanic vs non-Hispanic), region (Asia and Latin America vs other), and BMI class (normal [< 25 kg/m^2] or overweight [≥ 25 to < 30 kg/m^2] vs obese [≤ 30 kg/m^2]); and three HIV disease-specific factors: CD4 count (< 200 vs ≥ 200 cells/µl), HIV-1 RNA (≤ 100,000 vs > 100,000 c/ml), and HIV disease status (asymptomatic vs symptomatic or AIDS). The significance level for model entry and retention was specified as 0.2 and 0.05, respectively. The multivariate model at each postbaseline visit ultimately included eight of these risk factors (CD4 count, HIV-1 RNA, HIV disease status, age, race, ethnicity, region, and BMI class).

Multivariate regression model for absolute weight

Stepwise model selection was used to identify baseline risk factors associated with absolute weight using linear regression models among all visits, which included eight baseline risk factors. The baseline risk factors considered for inclusion were five traditional obesity risk factors: age (< 50 vs ≥ 50 years), sex at birth (male vs female), race (Black vs non-Black), ethnicity (Hispanic vs non-Hispanic), and region (Asia and Latin America vs other); and three HIV disease-specific factors: CD4 count (< 200 vs ≥ 200 cells/µl), HIV-1 RNA (≤ 100,000 vs > 100,000 c/ml), and HIV disease status (asymptomatic vs symptomatic or AIDS). The significance level for model entry and retention was specified as 0.2 and 0.05, respectively.

The multivariate model at each visit (including baseline) ultimately included seven of these risk factors (CD4 count, HIV-1 RNA, HIV disease status, age, race, ethnicity, and region).

Supplemental Table 1 Baseline characteristics of participants in Study 1489 and Study 1490 (Randomized Phase).

	Study 1489		Study 1490	
	**B/F/TAF (n = 314)**	**DTG/ABC/3TC (n = 315)**	**B/F/TAF (n = 320[a])**	**DTG + F/TAF (n = 325)**
Age, years, median (range)	31 (18–71)	32 (18–68)	33 (18–71)	34 (18–77)
Male sex at birth, n (%)	285 (91)	282 (90)	280 (88)	288 (89)
Race,[b] n (%)				
White	180 (58)	179 (57)	183 (57)	195 (60)
Black	114 (37)	112 (36)	97 (30)	100 (31)
Other[c]	12 (4)	14 (4)	33 (10)	20 (6)
Asian	6 (2)	10 (3)	7 (2)	10 (3)
Ethnicity, n (%)				
Hispanic/Latinx	72 (23)	65 (21)	83 (26)	81 (25)
HIV-1 RNA, $\log_{10}$ c/ml, median (Q1, Q3)	4.4 (4.0, 4.9)	4.5 (4.0, 4.9)	4.4 (4.0, 4.9)	4.5 (4.0, 4.8)
CD4 count, cells/µl, median (Q1, Q3)	443 (299, 590)	450 (324, 608)	440 (289, 591)	441 (297, 597)
Asymptomatic HIV infection, n (%)	286 (91)	286 (91)	286 (89)	288 (89)
$eGFR_{CG}$, ml/min, median (Q1, Q3)	126 (108, 146)	123 (107, 144)	120 (101, 142)	121 (103, 145)
Weight, kg, median (Q1, Q3)	77 (68, 88)	78 (68, 90)	76 (68, 87)	76 (67, 89)
BMI, kg/m^2, median (Q1, Q3)	25 (22, 29)	25 (23, 29)	25 (22, 28)	25 (22, 28)
Diabetes mellitus, n (%)	19 (6)	9 (3)	22 (7)	22 (7)
Hypertension, n (%)	35 (11)	41 (13)	59 (18)	62 (19)

Percentages may not add up to 100% due to rounding.

[a]Postbaseline data were unavailable for six participants.
[b]Race was not permitted to be disclosed for two participants in the B/F/TAF group (Study 1489); these participants were excluded from percentage calculations.
[c]"Other" race consists of American Indian or Alaska Native, Native Hawaiian or Pacific Isander, or other.

3TC, lamivudine; ABC, abacavir; B, bictegravir; BMI, body mass index; c, copies; DTG, dolutegravir; F, emtricitabine; $eGFR_{CG}$, estimated glomerular filtration rate according to the Cockcroft–Gault formula; Q, quartile; TAF, tenofovir alafenamide.

Supplemental Table 2 Baseline characteristics of the pooled B/F/TAF groups, stratified by baseline viral load and CD4 count.

	**VL ≤ 100,000 c/ml (n = 515)**	**VL > 100,000 c/ml (n = 119)**	**CD4 count ≥ 200 cells/µl (n = 554)**	**CD4 count < 200 cells/µl (n = 80)**	**VL > 100,000 c/ml and CD4 count < 200 cells/µl (n = 39)**
Age, years, median (range)	32 (18–71)	33 (18–71)	31 (18–71)	36 (22–64)	36 (22–64)
Male sex at birth, n (%)	451 (88)	114 (96)	495 (89)	70 (88)	38 (97)
Race,[a] n (%)					
White	294 (57)	69 (58)	330 (60)	33 (42)	16 (42)
Black	169 (33)	42 (36)	174 (32)	37 (47)	18 (47)
Other[b]	41 (8)	4 (3)	39 (7)	6 (8)	2 (5)
Asian	10 (2)	3 (3)	10 (2)	3 (4)	2 (5)
Ethnicity, n (%)					
Hispanic/Latinx	137 (27)	18 (15)	139 (25)	16 (20)	4 (10)
Weight, kg, median (Q1, Q3)	77.5 (68.2, 89.2)	74.8 (66.7, 84.8)	77.2 (68.2, 88.0)	71.5 (64.5, 84.7)	71.9 (63.5, 85.3)
BMI, kg/m^2, median (Q1, Q3)	25.3 (22.4, 29.0)	24.0 (21.7, 26.7)	25.2 (22.4, 28.8)	24.1 (21.2, 26.5)	24.0(21.2, 25.9)
Asymptomatic HIV infection, n (%)	481 (93)	91 (76)	532 (96)	40 (50)	16 (41)
eGFR$_{CG}$, ml/min, median (Q1, Q3)	122 (105, 144)	122 (101, 143)	123 (105, 144)	118 (95, 136)	122 (95, 142)

[a]Race was not permitted to be disclosed for one participant from each category; these participants were excluded from percentage calculations.
[b]"Other" race consists of American Indian or Alaska Native, Native Hawaiian or Pacific Isander, or other.

B, bictegravir; BMI, body mass index; eGFR$_{CG}$, estimated glomerular filtration rate according to the Cockcroft–Gault formula; F, emtricitabine; Q, quartile; TAF, tenofovir alafenamide; VL, viral load.

Supplemental Table 3 Effect of baseline characteristics on weight change from baseline at Weeks 48–240 (pooled B/F/TAF groups).

**Variable**	**Estimated mean (95% CI) difference in weight change from baseline, kg**<br>***p* value**				
	**Week**				
	**48**	**96**	**144**	**192**	**240**
BMI category (overweight versus underweight/normal)	0.9 (−0.2, 2.0)<br>0.110	1.7 (0.3, 3.0)<br>0.018	1.2 (−0.5, 2.8)<br>0.168	0.6 (−1.3, 2.5)<br>0.517	0.3 (−1.8, 2.3)<br>0.801
BMI category (obese versus underweight/normal)	2.2 (0.9, 3.5)<br>0.001	3.0 (1.3, 4.7)<br>< 0.001	2.7 (0.6, 4.7)<br>0.011	1.6 (−0.7, 3.8)<br>0.166	2.0 (−0.4, 4.4)<br>0.102
Ethnicity (Non-Hispanic versus Hispanic)	0.6 (−0.7, 1.9)<br>0.367	0.3 (−1.3, 1.9)<br>0.686	0.03 (−1.9, 2.0)<br>0.974	0.6 (−1.5, 2.8)<br>0.568	1.0 (−1.4, 3.3)<br>0.418
Race (Black versus non-Black)	1.1 (−0.04, 2.2)<br>0.584	1.1 (−0.3, 2.5)<br>0.119	1.5 (−0.2, 3.1)<br>0.088	0.8 (−1.1, 2.7)<br>0.408	0.5 (−1.6, 2.6)<br>0.634
Age (< 50 versus ≥ 50 years)	1.3 (−0.1, 2.6)<br>0.069	1.4 (−0.3, 3.1)<br>0.115	0.6 (−1.4, 2.7)<br>0.547	1.1 (−1.3, 3.4)<br>0.370	2.4 (−0.1, 5.0)<br>0.061
Region (Asia and Latin America versus other)	0.5 (−1.9, 2.9)<br>0.069	2.6 (−0.5, 5.7)<br>0.096	3.2 (−0.5, 7.0)<br>0.091	2.9 (−1.1, 7.0)<br>0.158	2.6 (−1.7, 6.8)<br>0.23
CD4 count (< 200 versus ≥ 200 cells/µl)	4.9 (3.2, 6.6)<br>< 0.001	5.5 (3.3, 7.7)<br>< 0.001	5.4 (2.8, 8.1)<br>< 0.001	5.0 (2.1, 7.9)<br>< 0.001	4.3 (1.1, 7.6)<br>0.009
HIV-1 RNA (> 100,000 versus ≤ 100,000 c/ml)	1.2 (−0.2, 2.5)<br>0.082	2.1 (0.4, 3.7)<br>0.014	2.7 (0.7, 4.7)<br>0.008	2.6 (0.4, 4.8)<br>0.019	3.9 (1.5, 6.3)<br>0.002
HIV disease status (AIDS or symptomatic versus asymptomatic)	2.2 (0.3, 4.1)<br>0.026	2.4 (−0.03, 4.8)<br>0.053	1.1 (−1.9, 4.0)<br>0.474	1.6 (−1.5, 4.8)<br>0.311	2.5 (−1.1, 6.1)<br>0.170

Mean differences in weight change, 95% CIs, and *p* values were derived from a multivariate linear regression model for weight change at each timepoint (Week 48 to Week 240). Bold font indicates statistical significance ($p < 0.05$). Baseline value was defined as the last nonmissing value obtained on or prior to the first dose of the study treatment (B/F/TAF).

B, bictegravir; BMI, body mass index; c, copies; CI, confidence interval; F, emtricitabine; TAF, tenofovir alafenamide.

Supplemental Table 4 Risk factors associated with ≥ 10% weight gain at Week 240 (pooled B/F/TAF groups).

**Variable**	**Odds ratio (95% CI)**<br>***p* value**
Age (< 50 vs ≥ 50 years)	1.79 (0.99, 3.25)<br>0.055
BMI category (underweight/normal vs overweight)	1.73 (1.08, 2.76)<br>0.021
BMI category (underweight/normal vs obese)	1.11 (0.65, 1.89)<br>0.700
CD4 count (< 200 vs ≥ 200 cells/µl)	2.90 (1.51, 5.56)<br>0.001
HIV-1 RNA (> 100,000 vs ≤ 100,000 c/ml)	2.10 (1.24, 3.57)<br>0.006

Odds ratios were based on a logistic regression model that included baseline CD4 count, HIV-1 RNA, age, and sex as risk factors. Bold font indicates statistical significance ($p < 0.05$). Includes participants with weight data at baseline and Week 240.

B, bictegravir; BMI, body mass index; c, copies; CI, confidence interval; F, emtricitabine; TAF, tenofovir alafenamide.

Supplemental Figure 1 Study design.

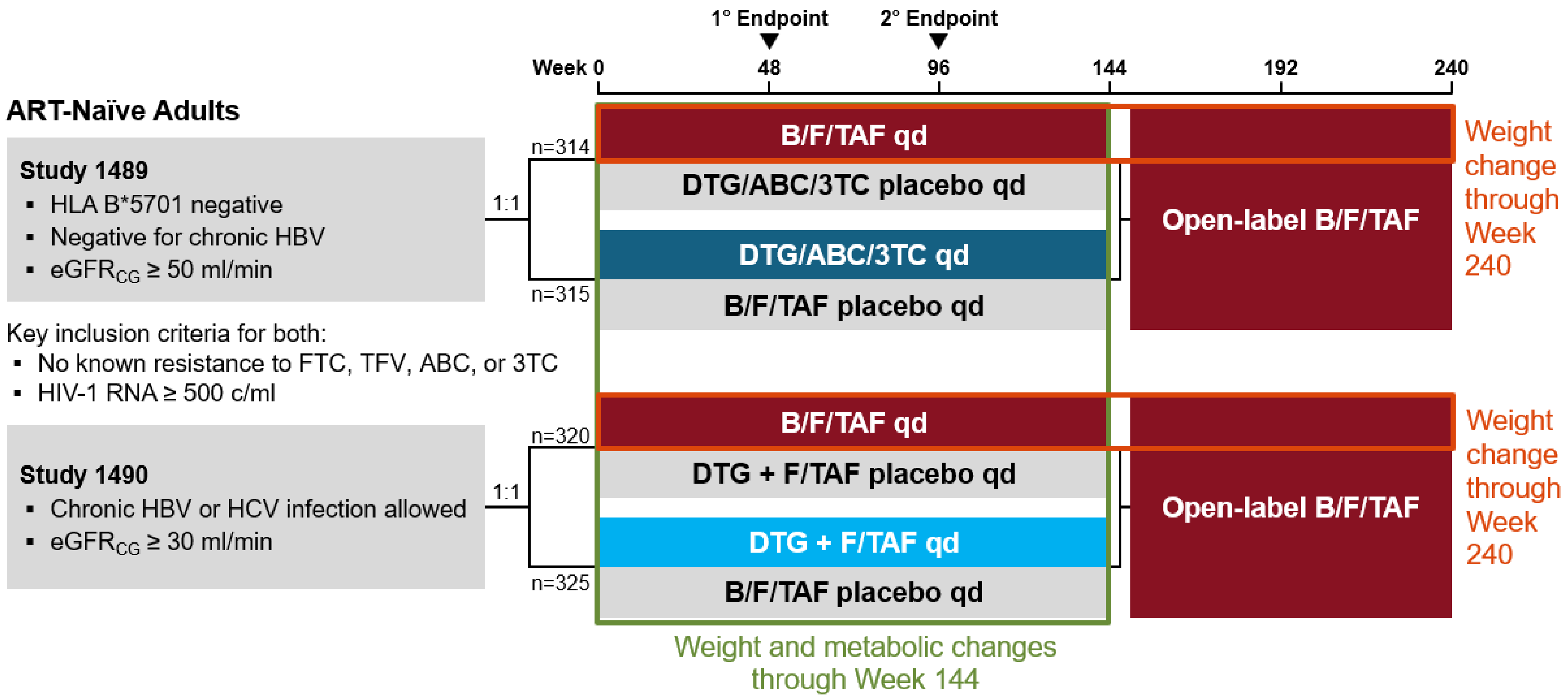

3TC, lamivudine; ABC, abacavir; B, bictegravir; c, copies; DTG, dolutegravir; eGFR$_{CG}$, estimated glomerular filtration rate according to the Cockcroft–Gault formula; F, emtricitabine; FTC, emtricitabine; HBV, hepatitis B virus; HCV, hepatitis C virus; HLA, human leukocyte antigen; qd, once daily; TAF, tenofovir alafenamide; TFV, tenofovir.

Supplemental Figure 2 Participant disposition through Week 240 for (a) Study 1489 and (b) Study 1490.

(a)

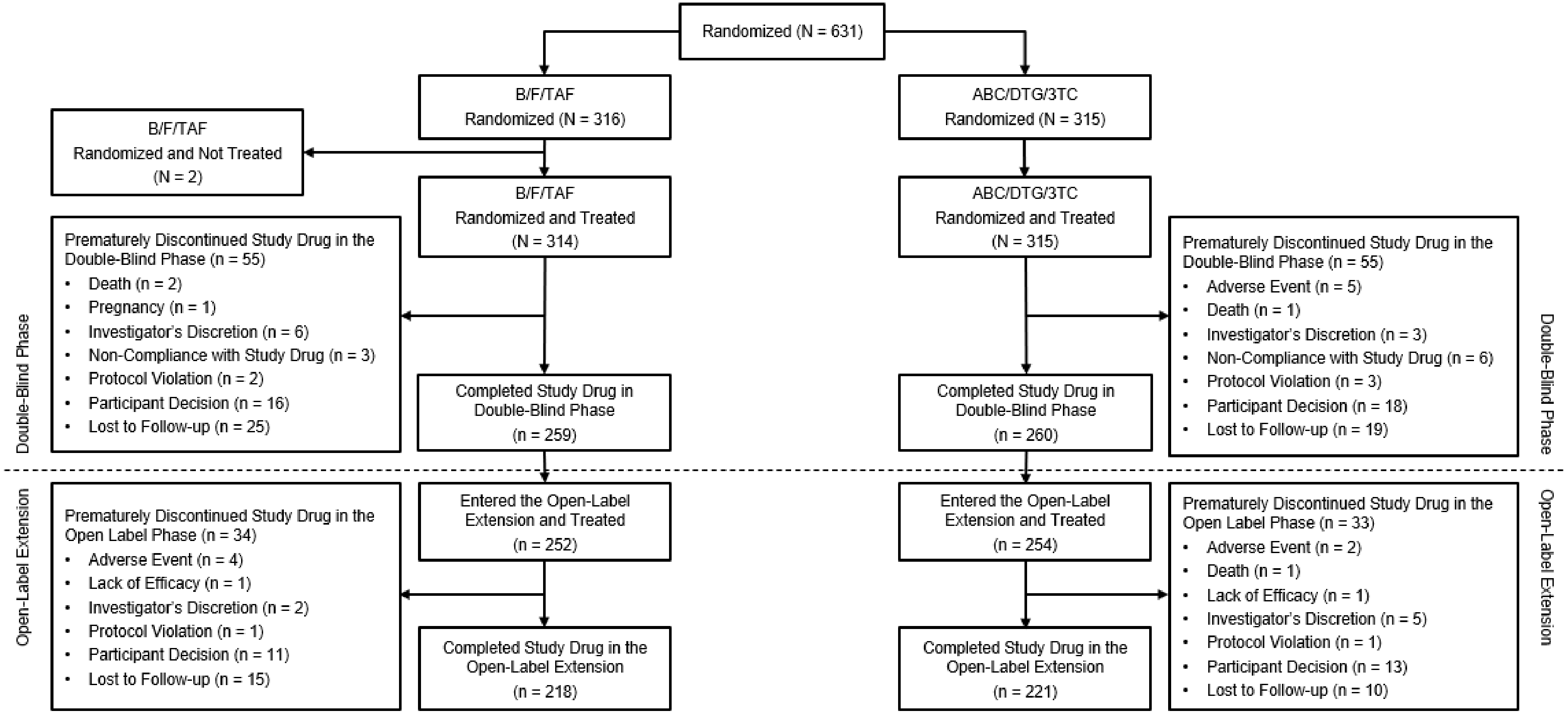

(b)

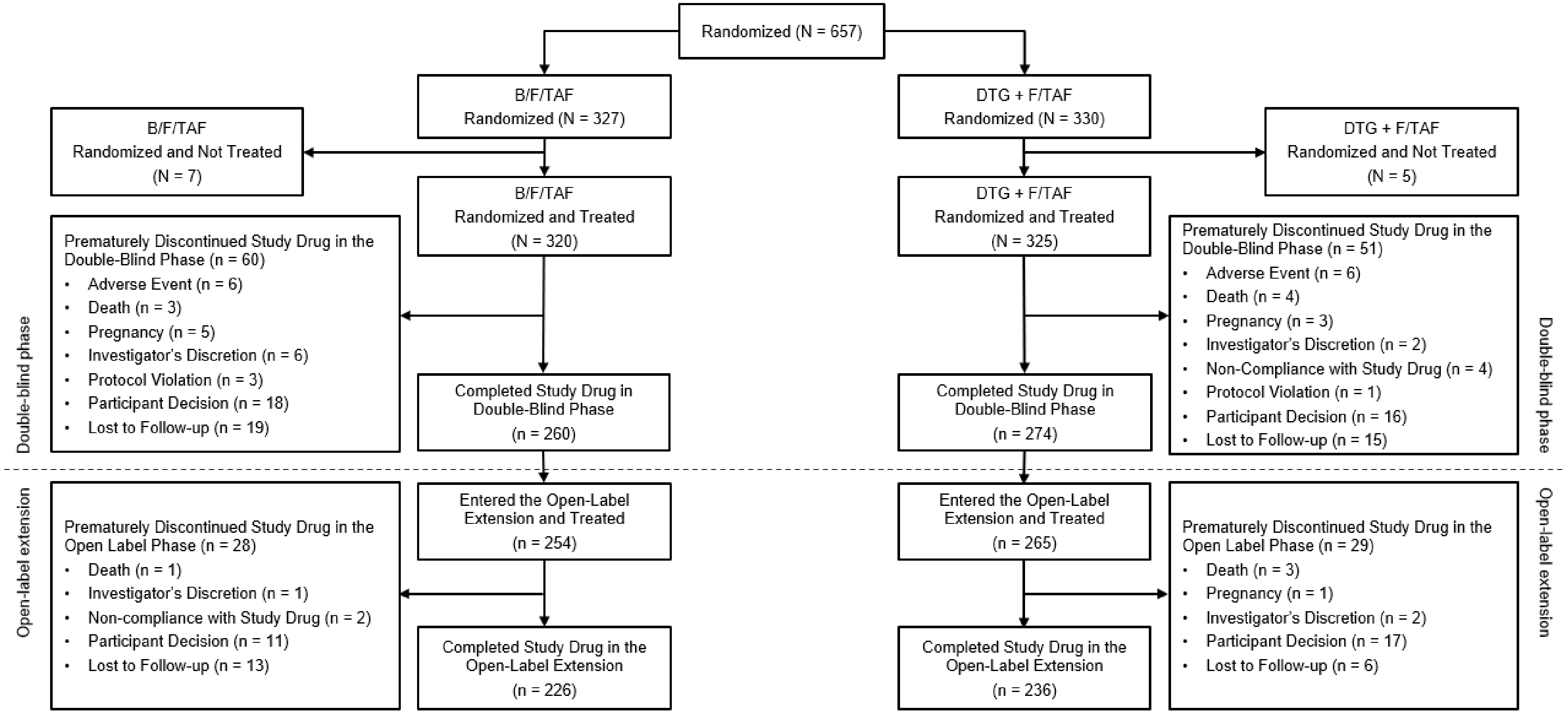

3TC, lamivudine; ABC, abacavir; B, bictegravir; DTG, dolutegravir; F, emtricitabine; TAF, tenofovir alafenamide.

Supplemental Figure 3 Fasting blood glucose levels through Week 144 in (a) Study 1489 and (b) Study 1490.

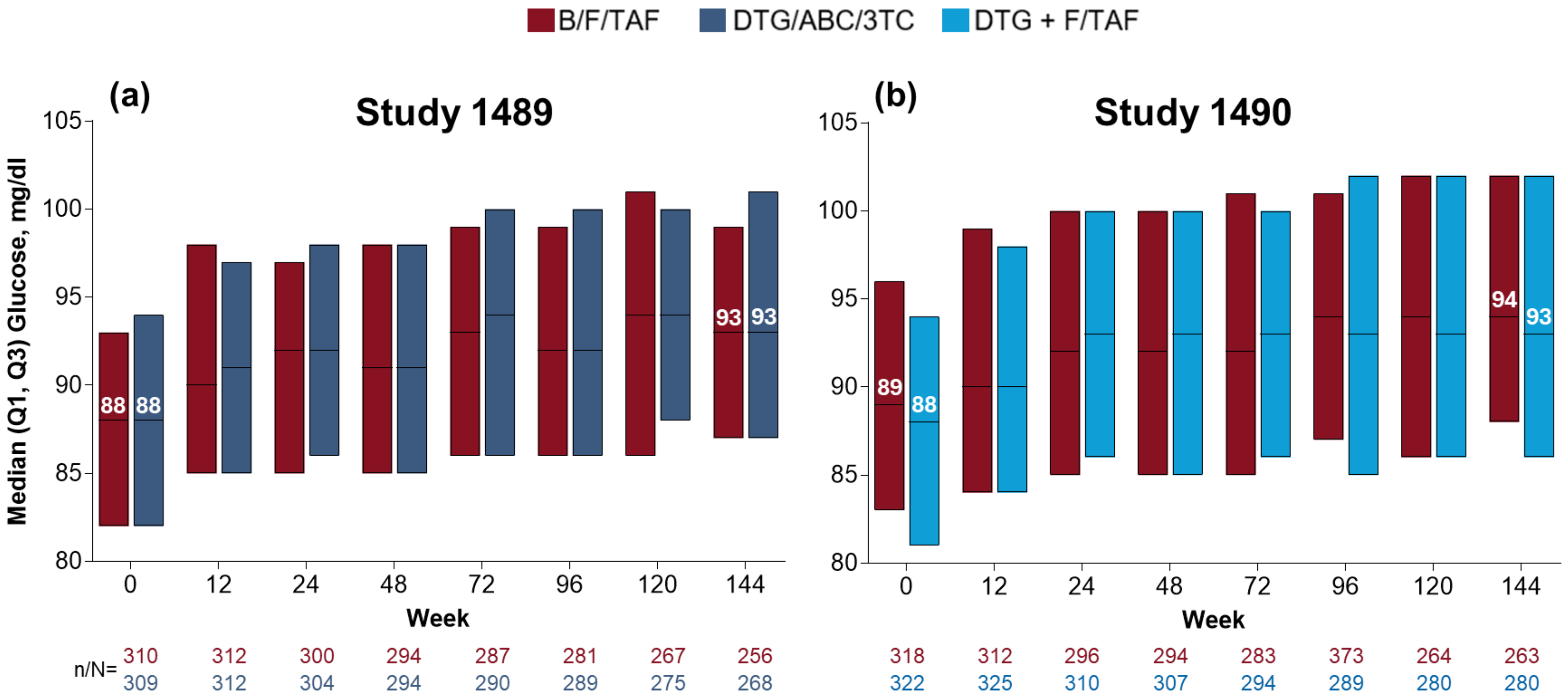

Includes participants with diabetes at baseline.

3TC, lamivudine; ABC, abacavir; B, bictegravir; DTG, dolutegravir; F, emtricitabine; Q, quartile; TAF, tenofovir alafenamide

Supplemental Figure 4 Proportions of participants experiencing graded treatment-emergent fasting lipid abnormalities (any grade) through Week 144 in (a) Study 1489 and (b) Study 1490.

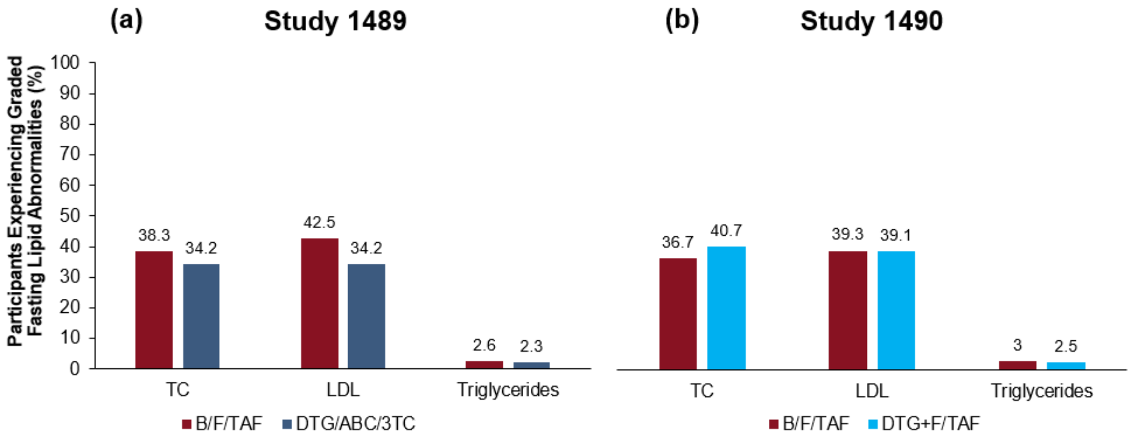

3TC, lamivudine; ABC, abacavir; B, bictegravir; DTG, dolutegravir; F, emtricitabine; LDL, low-density lipoprotein cholesterol; TAF, tenofovir alafenamide; TC, total cholesterol.

Supplemental Figure 5 Proportion of participants with HIV-1 RNA < 50 c/ml through Week 240 according to baseline VL and CD4 count: (a) missing = excluded, (b) missing = failure (pooled B/F/TAF groups).

(a)

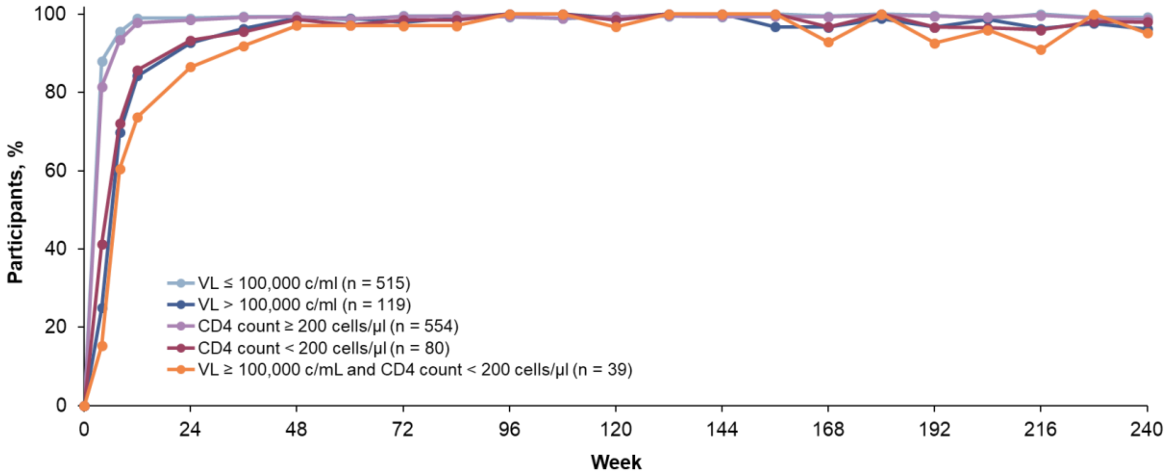

(b)

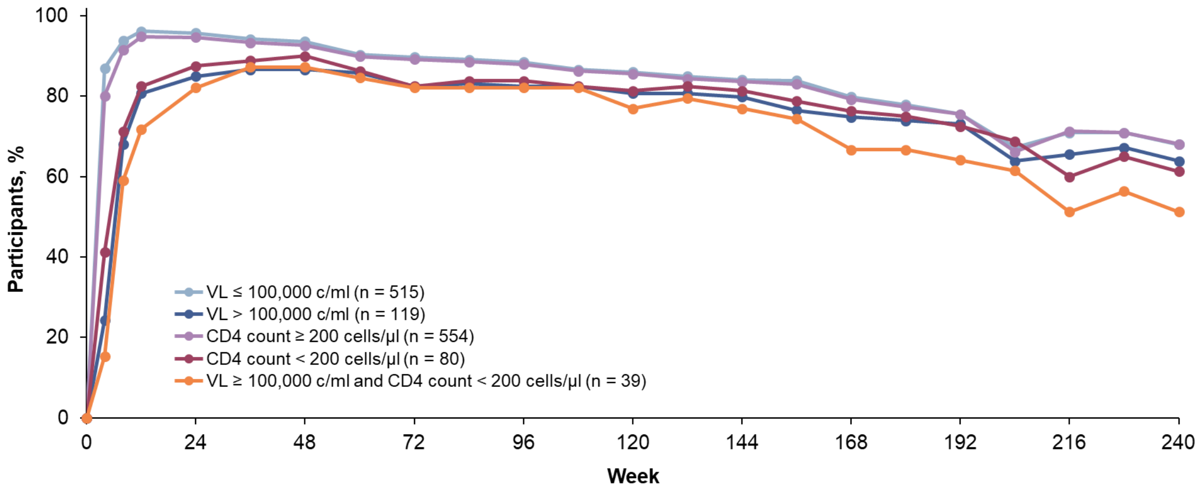

B, bictegravir; c, copies; F, emtricitabine; TAF, tenofovir alafenamide; VL, viral load.

Supplemental Figure 6 Change from baseline in CD4 count through Week 240 (pooled B/F/TAF groups).

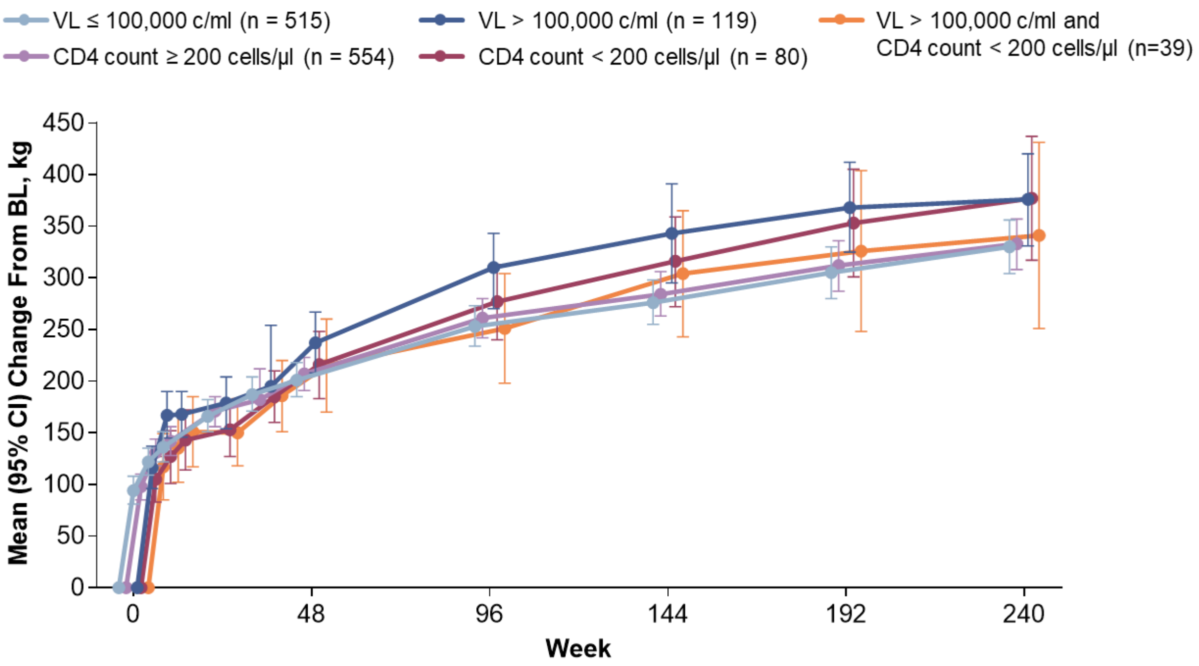

B, bictegravir; BL, baseline; c, copies; CI, confidence interval; F, emtricitabine; TAF, tenofovir alafenamide; VL, viral load.
